# The development of a novel natural language processing tool to identify pediatric chest radiograph reports with pneumonia

**DOI:** 10.3389/fdgth.2023.1104604

**Published:** 2023-02-22

**Authors:** Nancy Rixe, Adam Frisch, Zhendong Wang, Judith M. Martin, Srinivasan Suresh, Todd A. Florin, Sriram Ramgopal

**Affiliations:** ^1^ Division of Pediatric Emergency Medicine, UPMC Children’s Hospital of Pittsburgh, University of Pittsburgh School of Medicine, Pittsburgh, PA, United States; ^2^ Department of Emergency Medicine, University of Pittsburgh School of Medicine, Pittsburgh, PA, United States; ^3^ School of Computing and Information, University of Pittsburgh, Pittsburgh, PA, United States; ^4^ Division of General Academic Pediatrics, UPMC Children’s Hospital of Pittsburgh, University of Pittsburgh School of Medicine, Pittsburgh, PA, United States; ^5^ Division of Health Informatics, UPMC Children’s Hospital of Pittsburgh, University of Pittsburgh School of Medicine, Pittsburgh, PA, United States; ^6^ Division of Emergency Medicine, Department of Pediatrics, Ann & Robert H. Lurie Children's Hospital of Chicago, Northwestern University Feinberg School of Medicine, Chicago, IL, United States

**Keywords:** chest radiograph, clinical decision support, machine learning, natural language processing, pediatric, pneumonia

## Abstract

**Objective:**

Chest radiographs are frequently used to diagnose community-acquired pneumonia (CAP) for children in the acute care setting. Natural language processing (NLP)-based tools may be incorporated into the electronic health record and combined with other clinical data to develop meaningful clinical decision support tools for this common pediatric infection. We sought to develop and internally validate NLP algorithms to identify pediatric chest radiograph (CXR) reports with pneumonia.

**Materials and methods:**

We performed a retrospective study of encounters for patients from six pediatric hospitals over a 3-year period. We utilized six NLP techniques: word embedding, support vector machines, extreme gradient boosting (XGBoost), light gradient boosting machines Naïve Bayes and logistic regression. We evaluated their performance of each model from a validation sample of 1,350 chest radiographs developed as a stratified random sample of 35% admitted and 65% discharged patients when both using expert consensus and diagnosis codes.

**Results:**

Of 172,662 encounters in the derivation sample, 15.6% had a discharge diagnosis of pneumonia in a primary or secondary position. The median patient age in the derivation sample was 3.7 years (interquartile range, 1.4–9.5 years). In the validation sample, 185/1350 (13.8%) and 205/1350 (15.3%) were classified as pneumonia by content experts and by diagnosis codes, respectively. Compared to content experts, Naïve Bayes had the highest sensitivity (93.5%) and XGBoost had the highest F1 score (72.4). Compared to a diagnosis code of pneumonia, the highest sensitivity was again with the Naïve Bayes (80.1%), and the highest F1 score was with the support vector machine (53.0%).

**Conclusion:**

NLP algorithms can accurately identify pediatric pneumonia from radiography reports. Following external validation and implementation into the electronic health record, these algorithms can facilitate clinical decision support and inform large database research.

## 1. Introduction

Pneumonia is a significant cause of morbidity among children, resulting in a large proportion of unscheduled healthcare visits worldwide (1). In the United States, pneumonia accounts for 1%–4% of all emergency department (ED) visits in children and leads to greater than 100,000 hospitalizations annually (2–5). Among patients in children’s hospitals with possible pneumonia, greater than 80% receive a chest radiograph (CXR) which are frequently used, in addition to clinical presentation, to determine the need for antimicrobial therapy (6, 7). Previous studies have demonstrated wide variation in management strategies, including variable use of guideline-concordant antibiotics and inconsistent severity-adjusted hospitalization rates among this patient population (8–11). Electronic clinical decision support (CDS) tools have emerged as a way to align patient care with guideline-concordant therapy and management strategies (12–14). The utility of CDS tools in the pneumonia literature has been limited by the ability to incorporate free-text data, including CXR reports, into the electronic algorithm. Natural language processing (NLP), a class of machine learning which uses rule-based algorithms to convert unstructured text into encoded data, may overcome this limitation by interpreting and classifying large volumes of unstructured electronic text. Use of NLP in a comprehensive CDS tool that incorporates the chief complaint, historical data, vital signs and laboratory values, may allow for the rapid and accurate identification of disease, assist with guideline-concordant recommendations, and minimize unnecessary alert fatigue.

As the electronic health record (EHR) evolves, clinicians and researchers are increasingly able to query and utilize large volumes of electronic data to generate electronic CDS tools and to inform large dataset research. A CDS tool for pneumonia, for example, would take clinical data in combination with radiology data (such as CXRs and their interpretation) to calculate a predicted probability for this outcome. When this probability occurs within certain stakeholder-defined risk parameters, CDS tools may inform the clinician with respect to the best course of action (Supplementary Figure) (15). NLP offers a mechanism by which to rapidly interpret CXR reports and incorporate the encoded result into a CDS tool. The reported use of NLP in the pediatric pneumonia literature has been sparse, with prior studies limited to a small number of radiology reports and variable diagnostic performance (16, 17).

In this study, our objective was to develop and internally validate a novel NLP tool capable of rapidly identifying pediatric pneumonia from CXR reports with comparable accuracy to content experts and diagnosis codes.

## 2. Methods

We used a multicenter dataset of pediatric CXR reports to develop and internally validate multiple models capable of automatically identifying radiographic pneumonia based on radiologists’ interpretation. Model programming was performed using Python (version 3.9.1). Data management, calculation of Cohen’s Kappa coefficient, and assessment of model performance were performed in R (version 3.6.3; R Foundation for Statistical Computing, Vienna Austria).

### 2.1. Data source

**Figure 1:**
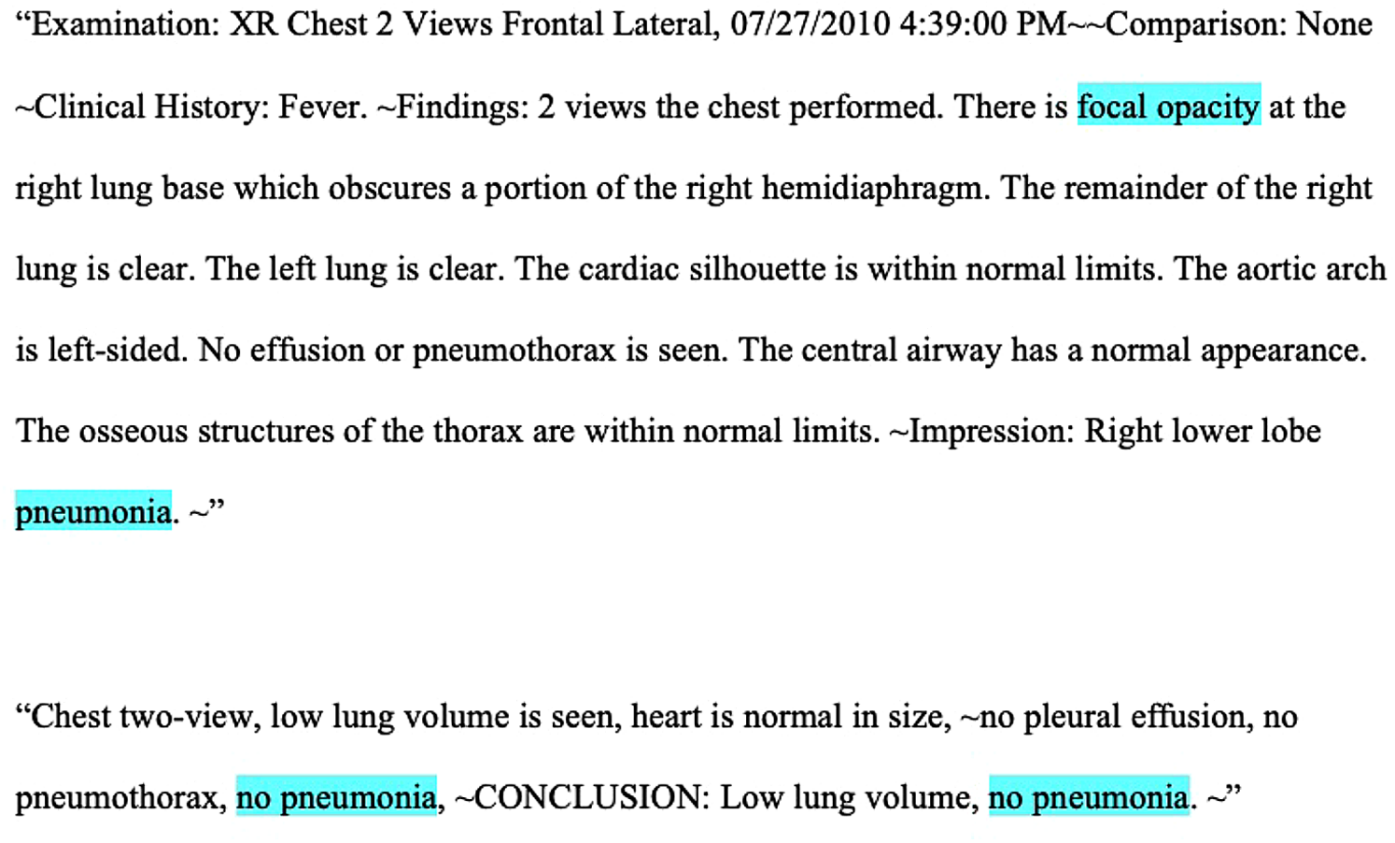
Highlighted examples of positive and negative chest radiograph reports, respectively.

We performed a retrospective study of pediatric CXR reports from the Pediatric Health Information System plus (PHIS+) database, a federated collection of clinical and administrative data from six large pediatric hospitals, collected between January 1, 2010 and December 31, 2012, and which has previously been employed for research using granular data not found within administrative datasets (18, 19). Figure 1 shows two examples of typical CXR reports. These generally consisted of an explanation of the image type, a clinical prompt, findings, and an interpretation, though exact formats varied. This study was deemed as exempt research by the investigators’ Institutional Review Boards.

### 2.2. Inclusion criteria

From the PHIS+ dataset, we identified all encounters for children ages 3 months to 18 years with a CXR performed in the ED and for which corresponding clinical data were identifiable within PHIS+. We only included children whose ED visits resulted in discharge or admission, thus excluding ambulatory or surgical encounters. For encounters with multiple CXRs during the same encounter, we retained the first. All types of CXR series were included, including portable, single-view, two-view, multiple-view, and foreign body aspiration series, regardless of the imaging study indication. Records with incomplete or missing CXR reports were excluded.

### 2.3. Data abstraction

For included encounters, we abstracted the attending radiologist CXR report and patient demographic data, including age, sex, race, ethnicity, hospital, and season of visit. We also abstracted relevant clinical characteristics, including complex chronic conditions (using a previously published diagnosis code-based classification system) (20), inpatient admission status, intensive care unit (ICU) status, need for extracorporeal membrane oxygenation (ECMO), need for mechanical ventilation, and mortality. Observation status was considered equivalent to inpatient status (21). These demographic data were not utilized in the development of the NLP tool but were provided to better describe the sample of pediatric patients with suspected CAP in the study population. We abstracted all (primary, associated and admission) discharge diagnosis codes.

### 2.4. Definition of pneumonia

In order to train the models, three content experts (NR, SR, AF) reviewed 200 randomly selected CXR reports and generated a mutually agreed upon list of the most frequently utilized keywords used to denote pneumonia (Table 1). Negative combinations of the keywords (e.g., “no infiltrate”) were also included to represent the absence of pneumonia. These keywords were then converted into tokens, (i.e., broken down into their most basic components) (Figure 1), and were then used to train models.

**Table 1:** Keywords used to train natural language processing algorithms.

Acute cardiopulmonary abnormality
Areas of pneumonia^a^
Clear lungs
Evidence of acute cardiopulmonary disease
Features of bacterial pneumonia
Focal airspace consolidation
Focal consolidation
Focal pulmonary infiltrate
Ground glass opacities
Hyperinflation^a^
Lungs are clear
Multifocal airspace disease^a^
Negative chest
No abnormality
No pleural effusion
Normal chest radiograph
Normal chest x-ray
Patchy consolidation
Perihilar opacities^a^
Pleural effusion
Pneumonia^a^
Pneumonia cannot be excluded
Reactive airway disease
Viral bronchiolitis
Within normal limits

^a^ Top 5 keywords in extreme gradient boosting model.

### 2.5. Derivation and validation sample

To assess the performance of the NLP modeling, we first retained a random sample of approximately 1% of the total number of included encounters to create the validation sample. This proportion was primarily selected due to the large total number of radiology reports included in the derivation sample which exceeded 170,000; the manual review of more than 1% of such a large number of chest x-ray reports was time and labor-prohibitive. To generate a validation sample, we performed stratified random sampling of equal proportions of chest radiographs positive and negative for pneumonia, which included approximately 1/3 from admitted patients to ensure similar disease acuity. Radiographs not used in the validation sample were retained for the derivation sample. For radiographs in the validation sample, two authors (NR and SR) independently reviewed and classified all CXR reports within the validation sample as either positive or negative for pneumonia based on clinical expertise. We calculated a Cohen's kappa coefficient to assess interrater reliability between the primary reviewers. A third, independent content expert (JR) reviewed the discrepant records and assigned a final code of pneumonia or no pneumonia. The remaining 99% of CXR reports that were not included in the validation dataset were included in the derivation sample.

### 2.6. Outcome measures

For the purposes of model validation, we used two outcomes: any diagnosis code of pneumonia (including the principal, admission, and any associated diagnosis) and diagnosis by expert consensus. A diagnosis of pneumonia was defined by an International Classification of Disease, 9th edition (ICD-9) discharge diagnosis code of pneumonia (Supplementary Table). While content expert annotation was considered more clinically applicable, we also evaluated its performance in the prediction of ICD-9 codes, as models were trained using these parameters. We used a previously validated list of diagnosis codes for CAP (4).

### 2.7. Development of natural language processing tool

To derive the NLP algorithms, we used six frequently employed NLP methods: word embedding, extreme gradient boosting (XGBoost), light gradient boosting machine (LightGBM), Support Vector Machine (SVM), Naïve Bayes, and logistic regression. We chose these representative algorithms on the basis of their prior use (Naïve Bayes, Support Vector Machines), frequent use in other domains (word embedding, logistic regression), and novelty (XGBoost and LightGBM).

#### 2.7.1. Word embedding

Word embedding refers to a classification technique in unsupervised machine learning that mathematically embeds a word or phrase from a space with potentially infinite meanings per word into a numerical vector space with fewer meanings in order to create the simplest numerical classification for complex language using an artificial neural network (22). Put another way, word embedding classifies many related words into simpler high dimensional vectors. For example, using word embedding, the words “happy” and “joyful” would be classified into the same numerical vector. We chose this machine learning technique, in part, because of its applicability to medicine, which often has many words that have similar meanings (for example, pneumonia, infiltrate, and consolidation).

**Figure 2:**
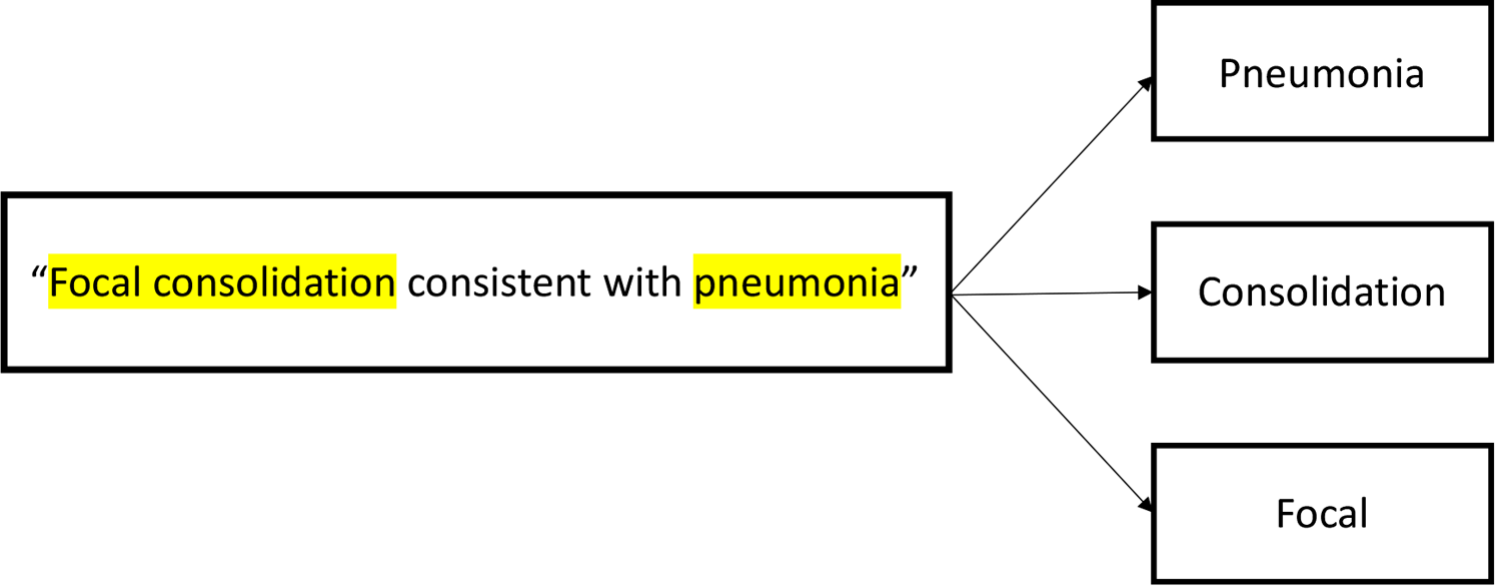
A simplified example of our skip-gram model.

We first trained specific word embedding on pneumonia-related clinical text. We then used sample phrases constructed by the authors for pneumonia. We converted these phrases into embedding by calculating the average word embedding for all tokens in a given phrase. Next, to apply word embedding to the dataset for classification, each CXR report was broken up into tokens. A skip-gram model, using a window of words of size 10 with a dimension of 100, was utilized to predict context words from the input, or target, words (Figure 2). A window of 10 is considered to be the reference standard size to train skip-gram models (24). We used 100 as the dimension of the vector because it was sufficient to capture an adequate amount of semantic information from words without requiring prolonged training time. The window of words with the highest cosine similarity score for each keyword was used to classify a CXR report as “pneumonia” or “no pneumonia.” We used the *Genism* Word2Vec library to train the word embedding from the given clinical documents (23).

#### 2.7.2. XGBoost and LightGBM

Gradient boosting is a form of meta-analytic machine learning that combines weak, individual prediction models to generate a more accurate aggregate target model that can perform regression and classification analyses. XGBoost is an open-source software designed to improve gradient boosting by performing parallelized decision tree building, tree-pruning using a depth-first approach and employing regularization to avoid overfitting the data. XGBoost was utilized primarily for its recent emergence as the optimal technique in many machine learning applications (24). From our training dataset, each keyword and its potentially negative expansion were used as initial inputs (24). We controlled the maximum depth of the tree as 3 and the learning rate as 0.3 based on the optimal model performance with these hyperparameters (25). Similarly, LightGBM is a gradient boosting framework based on decision tree algorithms which develops asymmetric trees, but differs from XGBoost through the development of more selective (e.g., “leaf-wise”) growth instead of level-wise growth. The LightGBM was set to have 31 leaves, and a learning rate of 0.05. As with word embedding, both gradient boosting techniques use an artificial neural network to calculate the embedding of the input text.

#### 2.7.3. SVM

**Figure 3:**
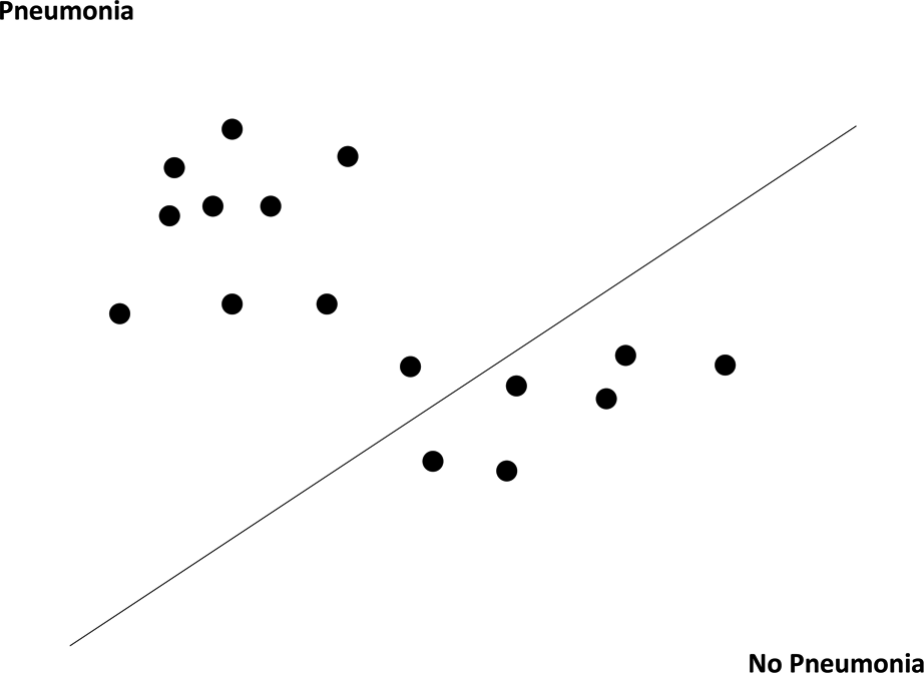
A simplified two-dimensional example of our linear kernel method.

SVM is a classification technique in supervised machine learning that maps data points (or support vectors) onto an N-dimensional hyperplane (where N equals the number of features) in order to classify them. For our SVM model, we implemented the “linear kernel” method, which classifies support vectors in a linear decision plane (Figure 3) (26, 27). Previous studies have utilized SVM to extract free text information from CXR reports with reasonable sensitivity and specificity (28).

#### 2.7.4. Naïve Bayes

Naïve Bayes is an NLP technique which relies on Bayes’ theorem and assumes independence of predictor variables when calculating the probability that predictor variables are related to the target variable (29). Naïve Bayes has served as a foundational NLP technique in studies examining both neonatal and adult pneumonia (16). For our Naïve Bayes model, we used the default configuration of a “Gaussian Naïve Bayes” model from the sklearn python package (30).

#### 2.7.5. Logistic regression

Logistic regression is commonly applied in machine learning contexts and uses Maximum Likelihood Estimation to classify the probability of a dichotomous outcome using predictor data. In NLP contexts, logistic regression is applied following feature extraction of vectorized text, similar to SVM and Naïve Bayes.

### 2.8. Statistical analysis

Study demographics and clinical characteristics were summarized using proportions for categorical variables and median and interquartile range for continuous variables. Characteristics of CXR reports that were positive versus negative for pneumonia were compared using *χ*^2^ tests for categorical variables and Wilcoxon rank sum for continuous variables. We compared demographic and clinical characteristics of encounters in the derivation and validation datasets. Differences were considered statistically significant at a *p* value of <0.05. In the validation dataset, we calculated the sensitivity and specificity of any ICD-9 diagnosis code of pneumonia compared to a reference standard of consensus content expert interpretation. For radiograph reports in the validation set, we calculated the unweighted Cohen's Kappa to measure inter-rater agreement for the manual CXR report review between the two primary reviewers. We calculated the performance of the four NLP models on both the derivation and validation datasets. For each of the machine learning techniques, we calculated the sensitivity (recall), specificity, positive predictive value (precision), negative predictive value, and positive and negative likelihood ratios with corresponding 95% confidence intervals (CI). Additionally, we calculated the accuracy and F1 score as measures of model performance frequently used in machine learning applications:

accuracy=(TP+TN)/(TP+TN+FP+FN)

F1=2x(precision×recall)/(precision+recall)

## 3. Results

### 3.1. Study inclusion

**Figure 4:**
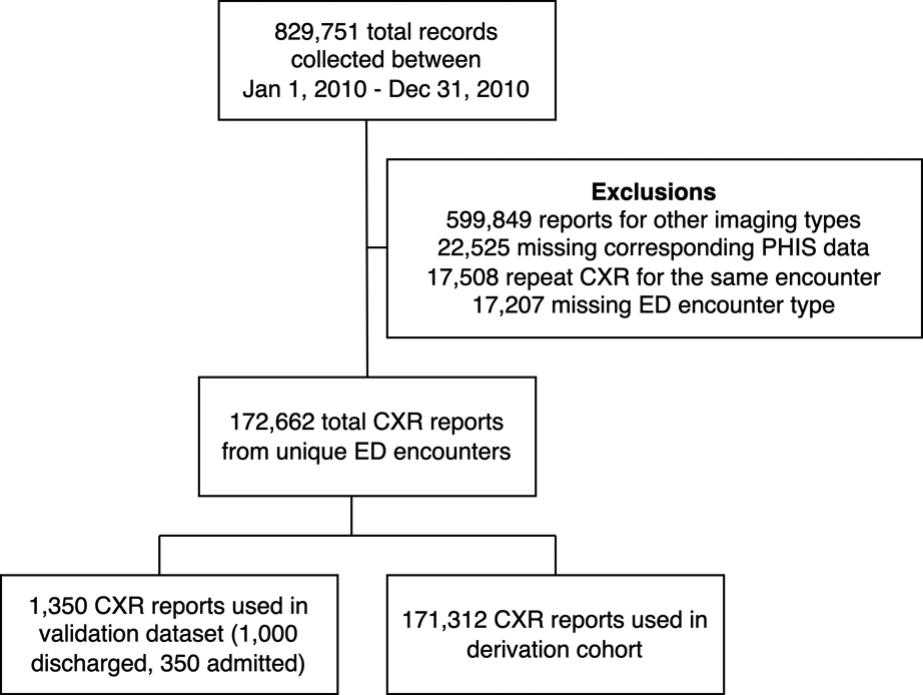
Study population.

A total of 829,751 encounters with imaging were collected from the six children’s hospitals within the PHIS+ database between January 1, 2010 and December 31, 2012. We excluded 599,849 (72%) imaging studies that were not CXRs, 22,525 (2.7%) that lacked a discharge diagnosis code, and 17,508 (2.1%) that were duplicate CXRs within the same encounter. 17,207 (2.1%) non-ED encounters were also excluded. A total of 172,662 (21%) CXR reports from unique ED encounters were included in the final analysis. This was divided into a derivation sample consisting of 171,312 (99.2%) CXR reports and a validation sample consisting of 1,350 (0.8%) CXR reports (Figure 4).

### 3.2. Descriptive data

The mean patient age for all included encounters was 3.7 years (1.6–8.7); 55% of encounters represented male patients. A median of 28,694 (IQR 19,280–39,943) CXR reports were collected from each hospital. 33,412 (19.3%) of encounters contained at least one diagnosis code of a complex chronic condition. A total of 27,105 (15.6%) encounters contained any ICD-9 diagnosis code of pneumonia and 20,493 (11.9%) encounters had a primary diagnosis code of pneumonia (Table 2).

**Table 2:** Demographics and clinical characteristics of all included encounters.

	Overall (*N* = 172,662)	No pneumonia diagnosis (*N* = 145,557)	Pneumonia diagnosis (*N* = 27,105)
**Demographics**			
Age, year, median (IQR)	3.7 (1.6–8.7)	3.7 (1.4–9.9)	3.7 (1.8–7.3)
Male, *n* (%)	95,245	80,796 (55.5%)	14,449 (53.3%)
Race, *n* (%)			
White	76,062	62,214 (44.1%)	11,848 (43.7%)
Black	48,044	40,961 (28.1%)	7,083 (26.1%)
Other or more than one	48,556	40,382 (27.7%)	8,174 (30.2%)
Ethnicity, *n* (%)			
Hispanic or latino	17,731	14,917 (10.2%)	2,814 (10.4%)
Non-hispanic or latino	127,664	107,664 (74.0%)	20,000 (73.8%)
Unknown	27,267	22,976 (15.8%)	4,291 (15.8%)
Hospital, *n* (%)*			
Hospital A	20,043	17,196 (11.8%)	2,847 (10.5%)
Hospital B	37,345	32,137 (22.1%)	5,208 (19.2%)
Hospital C	39,943	32,409 (22.3%)	7,534 (27.8%)
Hospital D	41,316	34,831 (23.9%)	6,485 (23.9%)
Hospital E	14,735	12,049 (8.3%)	2,686 (9.9%)
Hospital F	19,280	16,935 (11.6%)	2,345 (8.7%)
Month, *n* (%)			
Winter	53,706	44,134 (30.3%)	9,572 (35.3%)
Spring	39,074	33,432 (23.0%)	5,642 (20.8%)
Summer	32,971	28,968 (19.9%)	4,003 (14.8%)
Fall	46,911	39,023 (26.8%)	7,888 (29.1%)
**Clinical characteristics**			
Complex chronic condition, *n* (%)*	33,412	28,510 (19.1%)	4,902 (18.1%)
Admitted from ED, *n* (%)*	60,623	49,294 (33.9%)	11,329 (41.8%)
ICU admission, *n* (%)*	11,891	9,321 (6.4%)	2,570 (9.5%)
Use of ECMO, *n* (%)	83	64 (<0.1%)	19 (0.1%)
Use of mechanical ventilation, *n* (%)*	5,840	4,524 (3.1%)	1,316 (4.9%)
In-hospital mortality, *n* (%)	509	420 (0.3%)	89 (0.3%)

ED, emergency department; ECMO, extracorporeal membrane oxygenation; IQR, interquartile range; ICU, intensive care unit. Comparisons made using chi-squared or Wilcoxon rank-sum tests as appropriate.

* *p* < 0.05.

### 3.3. Validation sample

In the validation sample, 185/1,350 (13.7%) of CXR reports were classified by reviewers as positive for pneumonia. Concordance between reviewers demonstrated a kappa of 0.86. A total of 205 (15.3%) encounters had any ICD-9 diagnosis of pneumonia. 157 (11.6%) encounters had a primary ICD-9 diagnosis code of pneumonia (Table 3). There were statistically significant differences between encounters in the derivation and validation samples with regard to hospital location, complex chronic condition, admission from the ED, admission to the ICU, and the need for mechanical ventilation. There were no significant differences between in gender, race, ethnicity, need for ECMO, in-hospital mortality, or any primary ICD-9 diagnosis codes of pneumonia. When comparing the presence of an ICD-9 diagnosis code for pneumonia to annotation of radiographs by context experts (as the reference standard), ICD-9 diagnosis codes had a sensitivity of 93.3%, specificity of 69.2%, positive predictive value of 95.0%, and negative predictive value of 62.1%.

**Table 3:** Demographics and clinical characteristics of encounters in the derivation and validation samples.

	Derivation (*N* = 171,312)	Validation (*N* = 1,350)	*p* *
**Demographics**			
Age, year, median (IQR)	3.70 (1.43–9.46)	3.83 (1.40–9.59)	0.35
Male, *n* (%)	94,522 (55.2)	723 (52.6)	0.21
Race, *n* (%)			0.38
White	75,492 (44.1)	570 (42.2)	
Black	47,660 (27.8)	384 (28.4)	
Other or more than one	48,160 (28.1)	396 (29.3)	
Ethnicity, *n* (%)			0.62
Hispanic or latino	17,584 (10.3)	147 (10.9)	
Non-hispanic or latino	12,6681 (73.9)	983 (72.8)	
Unknown	27,047 (15.8)	220 (16.3)	
Hospital, *n* (%)			0.05
Hospital A	19,878 (11.6)	165 (12.2)	
Hospital B	37,080 (21.6)	265 (19.6)	
Hospital C	39,605 (23.1)	338 (25.0)	
Hospital D	40,985 (23.9)	331 (24.5)	
Hospital E	14,644 (8.5)	91 (6.7)	
Hospital F	19,120 (11.2)	160 (11.9)	
Month, *n* (%)			0.64
Winter	53,276 (31.1)	430 (31.9)	
Spring	38,782 (22.6)	292 (21.6)	
Summer	32,723 (19.1)	248 (18.4)	
Fall	46,531 (27.2)	380 (28.1)	
**Clinical characteristics**			
Complex chronic condition, *n* (%)	33,180 (19.4)	232 (17.2)	0.04
Admitted from ED, *n* (%)	60,273 (35.2)	350 (25.9)	<0.01
ICU admission, *n* (%)	11,818 (6.9)	73 (5.4)	0.04
Use of ECMO, *n* (%)	83 (0.0)	0 (0.0)	0.85
Use of mechanical ventilation, *n* (%)	5,810 (3.4)	30 (2.2)	0.02
In-hospital mortality, *n* (%)	506 (0.3)	3 (0.2)	0.81
**Outcome**			
Any ICD-9 diagnosis of pneumonia	26,899 (15.7)	205 (15.3)	0.68
Primary ICD-9 diagnosis of pneumonia	20,336 (11.9)	157 (11.6)	0.82

ICD, International Classification of Disease; ED, emergency department; ECMO, extracorporeal membrane oxygenation; IQR, interquartile range; ICU, intensive care unit.

* Comparisons made using chi-squared or Wilcoxon rank-sum tests as appropriate.

### 3.4. Model performance on validation sample

When evaluating model performance on the validation sample compared to manual review, the Naïve Bayes model had the highest sensitivity of 93.5% (Table 4). The XGBoost model had the highest specificity of 98.8%, positive predictive value of 89.0%, likelihood ratio of 50.8 and overall performance with an F1 score of 72.4. When evaluating the validation sample using an outcome of ICD-9 diagnosis codes of pneumonia, the performance of all models was lower (Table 5). The Word Embedding, SVM, XGBoost, and LightGBM models each retained a specificity greater than 90% when assessed with ICD-9 diagnosis codes.

**Table 4:** Model characteristics of the validation cohort based on manual review by three content experts.

Model	Sensitivity % (CI)	Specificity % (CI)	PPV % (CI)	NPV % (CI)	LR+ (CI)	LR− (CI)	Acc %	F1
Word embedding	19.5 (14.0–25.9)	95.1 (93.6–96.2)	38.7 (28.5–48.9)	88.1 (86.2–89.9)	3.91 (2.6–5.8)	0.85 (0.79–0.91)	84.7	25.9
XGBoost	61.1 (53.7–68.1)	98.8 (98.0–99.3)	89.0 (82.2–93.8)	94.1 (92.6–95.4)	50.8 (29.8–86.6)	0.39 (0.33–0.47)	93.6	72.4
SVM	56.2 (48.7–63.5)	98.1 (97.2–98.8)	82.5 (74.8–88.7)	93.4 (91.8–94.7)	30.0 (19.3–45.9)	0.45 (0.38–0.53)	92.4	66.9
Naïve Bayes	93.5 (88.9–96.6)	74.2 (71.6–76.7)	36.6 (32.2–41.1)	98.6 (97.6–99.3)	3.63 (3.27–4.03)	0.09 (0.05–0.15)	76.9	52.6
Logistic regression	5.4 (2.6–9.7)	95.5 (94.2–96.6)	16.1 (8.0–27.7)	86.4 (84.4–88.2)	1.21 (0.63–2.34)	0.99 (0.95–1.03)	83.1	8.1
LightGBM	26.5 (20.3–33.5)	99.8 (99.4–100)	96.1 (86.5–99.5)	89.5 (87.7–91.1)	154.28 (37.84–629.02)	0.74 (0.68–0.80)	90.0	41.5

SVM, support vector machine; sensitivity, or recall; PPV, positive predictive value, or precision; NPV, negative predictive value; LR+, positive likelihood ratio; LR−, negative likelihood ratio; Acc, or accuracy, is equal to (TP + TN)/(TP + TN + FP + FN); F score, or F1, is equal to 2x (precision x recall)/(precision + recall).

**Table 5:** Model characteristics of the validation cohort when using an outcome based on any ICD-9 diagnosis code of pneumonia.

Model	Sensitivity % (CI)	Specificity % (CI)	PPV % (CI)	NPV % (CI)	LR+ (CI)	LR− (CI)	Acc %	F1
Word embedding	15.0 (1.03–21.1)	94.6 (91.5–94.5)	26.4 (18.0–35.2)	87.1 (85.4–89.2)	2.2 (1.48–3.29)	0.91 (0.86–0.97)	82.4	20.7
XGBoost	42.2 (35.0–49.6)	96.5 (94.2–96.6)	60.0 (51.0–68.5)	91.2 (89.5–92.8)	9.45 (6.9–12.9)	0.61 (0.53–0.69)	88.2	52.3
SVM	42.7 (35.5–50.2)	95.6 (94.3–96.7)	60.8 (89.6–92.8)	91.3 (89.6–92.8)	9.75 (7.11–13.9)	0.60 (0.53–0.68)	88.4	53.0
Naïve Bayes	80.1 (73.5–85.5)	73.1 (70.0–86.0)	32.1 (27.9–36.6)	95.8 (94.3–97.1)	2.98 (2.64–3.35)	0.27 (0.2–0.37)	74.1	48.6
Logistic regression	4.9 (2.4–8.7)	88.2 (86.2–90.0)	6.9 (3.4–12.3)	83.7 (81.5–85.8)	0.41 (0.22–0.77)	1.08 (1.04–1.12)	75.5	5.7
LightGBM	20.0 (14.8–26.1)	99.1 (98.4–99.6)	80.4 (66.9–90.2)	87.4 (85.4–89.1)	22.88 (11.65–44.94)	0.81 (0.75–0.86)	87.1	32.0

SVM, support vector machine; sensitivity, or recall; PPV, positive predictive value, or precision; NPV, negative predictive value; LR+, positive likelihood ratio; LR−, negative likelihood ratio; Acc, or accuracy, is equal to (TP + TN)/(TP + TN + FP + FN); F score, or F1, is equal to 2x (precision x recall)/(precision + recall).

### 3.5. False positives and false negatives

Each NLP algorithm was subject to false positives and false negatives. Within the validation sample, the Naïve Bayes model demonstrated the lowest false positive rates of 6.5% and 19.1% when compared to expert consensus and ICD-9 codes, respectively. The LightGBM method demonstrated the lowest false negative rate of (0.2% when compared to expert consensus and 0.9% when compared to ICD-9 codes). There was no significant overlap of tokens that triggered false negative and false positive results between each of the models.

## 4. Discussion

In this investigation, we evaluated six NLP models to interpret pediatric CXRs with pneumonia. The Naïve Bayes model was the most sensitive and the XGBoost model was the most specific and had the overall best performance when compared to manual expert review. The incorporation of this type of machine learning tool into electronic CDS algorithms, in combination with other EHR data, carries the potential to augment the rapid identification of patients with pneumonia and to facilitate the real-time application of evidence-based clinical guidelines. In addition, this type of NLP tool may expedite large database research by reducing the time needed for manual review.

An accurate NLP model for the identification of pneumonia can be used in “non-knowledge” [or statistically/machine-learning derived (31)]-based CDS in order to decrease unnecessary variation in care, improve antibiotic stewardship, and improve prognostication. Used in conjunction with additional historical and clinical factors, a comprehensive CDS for pneumonia may be best able to identify patients for whom imaging may be indicated, followed by interpretation of radiograph findings in the context of clinical data in order to provide guidance with respect to antimicrobial utilization and ED disposition. Our findings are demonstrative of the utility of NLP-based interpretation of radiography reports to improve the evidence-based management of pediatric pneumonia. These expand upon prior research on the use of NLP for the identification of pediatric pneumonia in several ways, including in the use of a multicenter dataset, comparing multiple NLP algorithms, and evaluating two forms of internal validation (content experts and diagnosis codes). NLP results in optimal model performance when the quantity of the input data is large, which reduces the effect of noise, or unexplainable variation, in the data. Furthermore, dividing large volumes of input data into a training and test dataset mitigates this risk of over-fitting the model to the training dataset (32). Previous investigations into the role of NLP in the classification of pediatric CXR reports with pneumonia have used training datasets with several hundred CXR reports and, in some cases, used the same dataset for training and testing (16, 17, 33). Meystre, et al., used a sampling of 282 pediatric annotated CXR reports from the PHIS+ dataset using Textractor, a library included within the Apache Unstructured Information Management Architecture framework, to develop a model with a sensitivity of 52.7% and specificity of 96.6% (16). Mendonça, et al., evaluated the performance of a multimodular clinical NLP algorithm (called MedLEE) to assist in the interpretation of neonatal radiographs and reported a sensitivity of 71% and 99% (17). More recently, Smith, et al., used a random forest classifier trained using 10,000 chest radiographs performed among children hospitalized at a single institution and implemented it using a clinical decision support system (34). These models had high sensitivity (89.9%) and specificity (94.9%) during implementation and demonstrate the potential of these applications for identifying pneumonia, though its performance across clinical settings has not yet been reported. By training our models using more than 170,000 CXR reports from a multicenter database, we increase the scale of prior work by several orders of magnitude. In addition, we retained a separate validation dataset, which was interpreted by independent content experts with excellent concordance. Following external validation, our models may have potentially greater generalizability and applications to other populations.

Prior NLP studies of both adult and pediatric CXR reports have frequently relied on Bayesian methodology to determine the presence or absence of pneumonia (35, 36). While Bayesian logic is considered simple, fast and effective, it relies on the premise that all features in the dataset are both equally important and independent, a potentially inaccurate assumption in the highly variable interpretation of chest radiographs (37, 38). In this investigation, different NLP algorithms provided differing diagnostic accuracy. Importantly, the highest performing algorithm utilized XGBoost, a relatively novel machine learning technique which has been trialed in a wide variety of applications, functions well on multiple operating systems, supports all major programming languages and has consistently shown superior performance in comparison to other NLP methodologies (24).

The robust performance of our NLP models supports its incorporation into electronic CDS tools to efficiently integrate patient information with clinical guidelines. Implementation of and compliance with CDS tools varies widely between hospital systems, practice settings, EHRs and individual users (39). Factors associated with low utilization rates of CDS tools include complexity of the system and difficulty integrating the tool into the workflow of the EHR (40).

XGBoost has recently emerged as the one of the highest-performing NLP algorithms across a variety of machine learning platforms, currently making it the most widely applicable NLP technology. Not surprisingly, our XGBoost model demonstrated the best overall performance (as measured by the F1 score). The accuracy of our XGBoost model may be further improved by the incorporation of other structured elements within the EHR that are known to be associated with pneumonia (including age, fever, and hypoxemia) (41, 42). Used in combination with these clinical factors, the use of automated radiograph interpretation may allow for an improved diagnostic accuracy, decreasing both false alarms (i.e., false positives) and missed cases (i.e., false negatives). As such, this NLP model may offer a standardized approach to building and implementing CDS tools, with the goals of improving antimicrobial stewardship, risk severity prediction, and in reducing unnecessary hospitalizations. The successful deployment of these algorithms will depend on an engaged stakeholder base and should follow principles recently identified by Shortliffe, et al., including (a) transparent reasoning, (b) seamless integration, (c) intuitive utilization, (d) clinical relevance, (e) recognition of the expertise of the clinician, and (f) constructed on a sound evidence base (43).

Our results additionally support the use of NLP research to facilitate large database research. By automatically finding actionable insight within large and complex volumes of electronic health data, a robust NLP model provides a natural solution to time-intensive manual review usually required by large database research (44). All models demonstrated tradeoffs between sensitivity and specificity to varying extends which may impact the clinical applicability of these algorithms when embedded into a decision support system model. For example, the Naïve Bayes model, demonstrated the highest sensitivity at 93.5%, which is likely due to its reliance on simple probabilities. When trained with domain experts, we theorize that Naïve Bayes would be best utilized to accurately capture true positives while reducing the time burden associated with manually reviewing and classifying thousands of unstructured imaging reports. In contrast, the XG Boost model demonstrated a higher PPV, as reflected in its higher F1 score; this model also demonstrated extremely high specificity. In combination with a highly specific model like XGBoost, integration of the Naïve Bayes algorithm into the EHR could facilitate the rapid and accurate identification of pediatric patients with pneumonia for prospective enrollment in clinical studies and allow for the provision of automatic disease-specific management guidelines. Logistic regression demonstrated poor performance which favored the majority (e.g., negative) class, suggesting its limited potential to meaningfully identify true positives with correspondingly poor sensitivity and positive predictive value.

It is worth noting that each of the four models demonstrated superior measures of diagnostic accuracy when validated with domain experts versus ICD-9 diagnosis codes of pneumonia. As such, our findings likely reflect the inherent difference in specificity between screening for pneumonia based solely on a radiograph report and the impact of incorporating additional clinical data like vital signs, physical exam findings, laboratory results, comorbid conditions and response to initial treatments, all of which are integrated into a final diagnosis code. We surmise that incorporation of an XGBoost-based model into a cohesive CDS tool, in combination with other clinical electronic data, would potentially result in enhanced performance for specific clinical applications. Other applications of machine learning with radiography have focused on direct image interpretation. Within pediatric research, these applications have been trialed for the diagnosis of pneumonia using Näıve Bayes, SVM, K-nearest neighbor (45), and transfer learning techniques (46), among others (47). These algorithms may be combined with radiologist interpretation to potentially work together in a complementary fashion to improve the overall accuracy of the predictive model.

Our findings are subject several limitations. First, the PHIS+ database did not contain the actual radiographs to verify the radiologists’ interpretations. The interpretation of chest radiography is complex: prior work, for example, has demonstrated wide interrater variability between radiology assessments regarding infiltrate versus atelectasis on the interpretation of pediatric imaging (38, 40). Second, our manual review process was based solely on domain expert interpretation and did not utilize a standardized set of agreed upon words or phrases to dichotomize the CXR reports into positive or negative. Despite this, the two primary reviewers retained a high degree of inter-rater agreement, which was comparable to prior studies that used a predetermined set of words to define positive and negative reports (18). We believe our approach is more generalizable and accurately reflects the natural variation between providers when interpreting free text clinical data. Third, our validation dataset was small, containing 1% of the original sample. Given the large size of the derivation sample, this amounted to a total of 1,350 CXR reports, which remained a sizable sample. In addition, PHIS+ is primarily an administrative database, and, as such, does not include time-based data or clinical notes. Although we were able to take advantage of the large repository of radiological data within PHIS+, the lack of other clinical data (i.e., clinical notes, etc.) make the development of a deep learning-based tool less applicable and represents an area of future research. Finally, most CXR reports in both the training and test datasets were negative for pneumonia, reflecting the relative paucity of pneumonia in the pediatric population compared to viral lower respiratory tract disease. This imbalance in the training data, a commonly encountered phenomenon in healthcare-related machine learning applications, has the potential to artificially increase the precision and decrease the recall (48). Despite this, the F1, or overall performance, of our XGBoost model was robust indicating an acceptable balance between precision and recall. Future work should focus on external validation and enhancing the performance of this model through interactive learning, including manual review of the false positive and false negative reports in order to mitigate the effects of the skewed input data and optimize model performance.

In this investigation, we used supervised and unsupervised learning techniques to generate novel NLP algorithms capable of identifying pediatric CXR reports positive for pneumonia with comparable accuracy to content experts and robust measures of diagnostic accuracy. Our results suggest that, following external validation, such tools could be integrated into comprehensive electronic CDS systems to enhance the automatic identification of pediatric patients with radiographic pneumonia and to inform large database research. Future research is needed to refine the NLP algorithms in order to apply them to specific clinical settings.

## Data Availability

The original contributions presented in the study are included in the article/**Supplementary Material**, further inquiries can be directed to the corresponding author.
