# Supplementary material for: The development of a novel natural language processing tool to identify pediatric chest radiograph reports with pneumonia
Source: Front Digit Health. 2023 Feb 22;5:1104604. doi: 10.3389/fdgth.2023.1104604 (PMC9992200; doi:10.3389/fdgth.2023.1104604)
Supplement: Supplementary file 1 [file Table1.docx]

**Supplementary Table.** International Classification of Disease, 9^th^ (ICD-9) revision diagnosis codes used for community-acquired pneumonia.

| **ICD-9 code** | **Diagnosis** |
| --- | --- |
| 480.0 | Pneumonia due to adenovirus |
| 480.1 | Pneumonia due to respiratory syncytial virus |
| 480.2 | Pneumonia due to parainfluenza virus |
| 480.8 | Pneumonia due to other virus not elsewhere classified |
| 480.9 | Viral pneumonia, unspecified |
| 481 | Pneumococcal pneumonia [Streptococcus pneumoniae pneumonia] |
| 482.0 | Pneumonia due to Klebsiella pneumoniae |
| 482.30 | Pneumonia due to Streptococcus, unspecified |
| 482.41 | Methicillin susceptible pneumonia due to Staphylococcus aureus |
| 482.42 | Methicillin resistant pneumonia due to Staphylococcus aureus |
| 482.83 | Pneumonia due to other gram-negative bacteria |
| 482.89 | Pneumonia due to other specified bacteria |
| 482.9 | Bacterial pneumonia, unspecified |
| 483.8 | Pneumonia due to other specified organism |
| 484.3 | Pneumonia in whooping cough |
| 485 | Bronchopneumonia, organism unspecified |
| 486 | Pneumonia, organism unspecified |
| 487.0 | Influenza with pneumonia |
