# Supplementary figures and images for: The development of a novel natural language processing tool to identify pediatric chest radiograph reports with pneumonia
Source: Front Digit Health. 2023 Feb 22;5:1104604. doi: 10.3389/fdgth.2023.1104604 (PMC9992200; doi:10.3389/fdgth.2023.1104604)

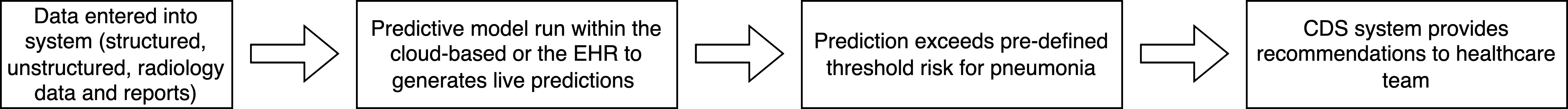

Supplement: Supplementary file 2 [file Image1.jpeg]
